# Secondary Evans Syndrome Presenting With Lupus Anticoagulant

**DOI:** 10.7759/cureus.63992

**Published:** 2024-07-06

**Authors:** Omar Abdelhalim, Claudia Serna, Nathalie Guerrero, Iris Onasis, Hazem Abosheaishaa

**Affiliations:** 1 Internal Medicine, Icahn School of Medicine at Mount Sinai, Queens Hospital Center, New York, USA; 2 Internal Medicine, The City University of New York School of Medicine, New York, USA; 3 Internal Medicine, St. George's University School of Medicine, St. George's, GRD; 4 Internal Medicine and Gastroenterology, Cairo University, Cairo, EGY

**Keywords:** autoimmune hemolytic anemia, deep vein thrombosis, immune thrombocytopenia, lupus anticoagulant, evans syndrome

## Abstract

Evans syndrome (ES), characterized by autoimmune hemolytic anemia (AIHA) and immune thrombocytopenia (ITP), often poses diagnostic challenges due to its varied etiology and clinical presentation. We present a case of secondary ES in a 41-year-old male with a history of AIHA and ITP, who presented with lower extremity erythema, warmth, and sensation of chest pressure. Initial laboratory investigations revealed thrombocytopenia, mild anemia, and a prolonged activated partial thromboplastin time (aPTT), prompting further evaluation. Subsequent testing revealed positive lupus anticoagulant (LA), anti-cardiolipin antibodies, and anti-beta-2-glycoprotein 1 antibodies, along with lower extremity deep vein thrombosis (DVT) and bilateral pulmonary embolism (PE). Treatment with therapeutic anticoagulation led to clinical improvement, highlighting the importance of recognizing hypercoagulable states in ES patients. This case underscores the significance of comprehensive differential diagnosis and timely intervention in optimizing outcomes for patients with ES.

## Introduction

Evans syndrome (ES) is an autoimmune condition that often manifests as two or more cytopenia, more commonly with autoimmune hemolytic anemia (AIHA) and immune thrombocytopenia (ITP) [1]. AIHA is an acquired immune disorder where the body produces autoantibodies against one’s own red blood cell (RBC) antigens resulting in hemolysis. Specifically, ES presents with warm AIHA, in which IgG antibodies react with RBC surface antigens at body temperature [1]. ITP purpura is another disorder of the immune system where the body produces antibodies directed against GPIIb/IIIa on the platelet surface. This immune-mediated platelet destruction results in thrombocytopenia. While primary ES is idiopathic and often a diagnosis of exclusion, secondary ES is believed to be due to other underlying autoimmune conditions, immunodeficiency disorders, or malignancy [2]. Although having a positive lupus anticoagulant (LA) has been observed in cases of ITP [3-5], the association between ES and positive LA syndrome has not yet been established. Here, we describe a case of secondary ES with initial blood work revealing positive LA.

## Case presentation

A 41-year-old male with a past medical history of ES presented with a 10-day history of erythema, redness, and warmth of his left calf with no history of trauma, fever, or shortness of breath. Nine months prior, the patient was admitted for symptomatic anemia and thrombocytopenia, in which he received eight units of blood and two units of platelets and was treated with intravenous immunoglobulin (IVIG), four cycles of rituximab, and five months of prednisone taper. Since then, the patient has been in remission and was not on any medications.

In the ED, the patient was in no acute distress, fully conscious, alert, and oriented. Examination of the lower extremities revealed a swollen and erythematous left lower leg from below the knee to above the ankle. There was no evidence of laceration, ecchymosis, erythematous streaking, or tenderness to palpation to bilateral lower extremities. Sensation remained intact throughout all extremities with strength 5/5. All other systemic examinations were unremarkable. Two hours after he arrived at the hospital, the patient reported a left-sided non-exertional, non-radiating chest pressure.

Initial lab investigations were done (Table 1) and revealed mild anemia, significant thrombocytopenia, and a prolonged activated partial thromboplastin time (aPTT). Due to the prolonged aPTT, subsequent mixing studies were ordered (Table 2) and failed to correct the aPTT time. Silica clotting time and dilute Russell's viper venom time (dRVVT) studies were positive indicating the presence of LA. Further investigations revealed positive anti-cardiolipin antibodies and positive anti-beta-2-glycoprotein 1 antibodies. The patient did not have any symptoms of active lupus and no prior history of any thrombotic event.

**Table 1:** The table shows initial investigations including complete blood count, basic metabolic pattern, hepatic function, and coagulation profile. CBC: complete blood count; WBC: white blood cell count; RBC: red blood cell count; HGB: hemoglobin; HCT: hematocrit; MCV: mean corpuscular volume; MCH: mean corpuscular hemoglobin; MCHC: mean corpuscular hemoglobin concentration; MPV: mean platelet volume; RDW: red cell distribution width; PLT: platelet count; Imm Gran %: immature granulocyte percentage; NRBC abs: nucleated red blood cell absolute count; NRBC %: nucleated red blood cell percentage; BUN: blood urea nitrogen; ALK PHOS: alkaline phosphatase; ALT (SGPT): alanine aminotransferase (serum glutamate pyruvate transaminase); AST (SGOT): aspartate aminotransferase (serum glutamate oxaloacetate transaminase); eGFR (cr): estimated glomerular filtration rate (based on creatinine); PT: prothrombin time; aPTT: activated partial thromboplastin time; INR: international normalized ratio

Components	Day 1	Day 2	Day 3	Reference Ranges
CBC				
WBC	5.53	5.44	5.95	4.80-10.80 x10^3^/mcL
RBC	4.57	4.62	4.61	4.70-6.10 x10^6^/mcL
HBG	13.2	13.1	13.4	14.0-18.0 g/dL
HCT	40.5	41.2	40.8	42.0-52.0%
MCV	88.6	89.2	88.5	80.0-99.0 fL
MCH	28.9	28.4	29.1	27.0-31.0 pg
MCHC	32.6	31.8	32.8	29.8-35.2 g/dL
MPV	10.2	10.1	9.2	8.7-12.9 fL
RDW	13.0	13.1	13.2	12.0-15.0%
PLT	95	98	110	150-400 x10^3^/mcL
Neutrophil %	65.6	58.8	62.7	44.0-70.0%
Lymphocyte %	21.2	23.9	21.8	20.0-45.0%
Monocyte %	7.2	8.1	8.1	2.0-45.0%
Eosinophil %	4.9	8.1	6.4	1.0-4.0%
Basophil %	0.4	0.4	0.5	0.0-2.0%
Imm Gran %	0.7	0.7	0.5	0.0-2.0%
Neutrophil abs	3.63	3.20	3.73	2.10-7.60 x10^3^/mcL
Lymphocyte abs	1.17	1.30	1.30	1.00-4.90 x10^3^/mcL
Monocyte abs	0.4	0.44	0.48	0.10-1.10 x10^3^/mcL
Eosinophils abs	0.27	0.44	0.38	0.10-0.40 x10^3^/mcL
Basophils abs	0.02	0.02	0.03	0.00-0.20 x10^3^/mcL
Immature gran abs	0.04	0.04	0.03	0.00-0.20 x10^3^/mcL
NRBC abs	0.00	0.00	0.00	≤0.00 x10^3^/mcL
NRBC %	0.0	0.0	0.0	0.0-0.0%
Serum				
Sodium	137	141	140	136-145 mmol/L
Potassium	3.9	3.8	4.1	3.5-5.1 mmoL/L
Chloride	103	103	102	98-108 mmol/L
CO_2_	27	24	28	22-29 mmol/L
BUN	19	15	16	6-23 mg/dL
Creatinine	0.82	1.05	1.08	0.70-1.20 mg/dL
Glucose	104	83	91	74-110 mg/dL
Calcium	9.0	8.7	8.8	8.6-10.3 mg/dL
Albumin	4.3	-	4.3	3.5-5.2 g/dL
Total protein	6.5	-	6.3	6.6-8.7 g/dL
Total bilirubin	0.50	-	0.6	0.00-1.20 mg/dL
ALK PHOS	86	-	82	40-129 U/L
ALT (SGPT)	20	-	20	0-41 U/L
AST (SGOT)	17	-	17	5-40 U/L
Anion gap	7	14	10	8-16 mEq/L
eGFR(cr)	>60	>60	>60	≥60 ml/min/1.73m^2^
Coagulation Studies				
PT	11.9	-	-	10.0-13.0 seconds
aPTT	61.9	-	-	25.1-36.5 seconds
INR	1.0	-	-	

**Table 2:** This table presents subsequent laboratory results focusing on the antiphospholipid antibody diagnostic panel. aPTT: activated partial thromboplastin time; NPP-PTT: normal pool plasma partial thromboplastin time; DRVVT: dilute Russell's viper venom time; SCT: silica clotting time; LA: lupus anticoagulant

Components	Values	Reference Ranges
aPTT 100%	55.6	24.5-35.6 seconds
NPP-PTT	33.6	24.5-35.6 seconds
aPTT 50/50	49.2	24.5-36.6 seconds
PTT 50/50 2H	52.7	24.5-36.6 seconds
dRVVT Interpretation	LA positive	-
Silica clotting time (SCT)	3.16	0.00-1.16 seconds
SCT interpretation	LA positive	-

**Figure 1:**
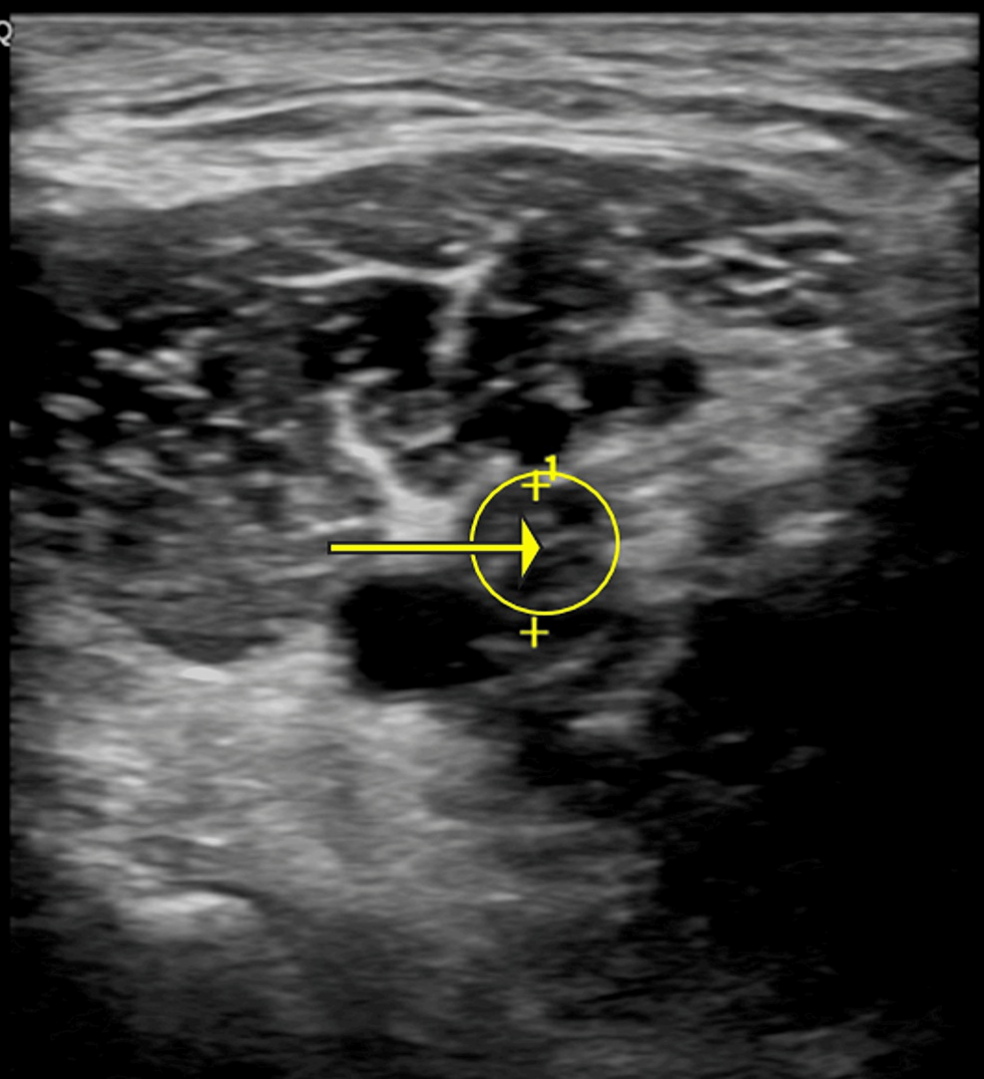
Non-compressible thrombus within a dilated left popliteal vein

**Figure 2:**
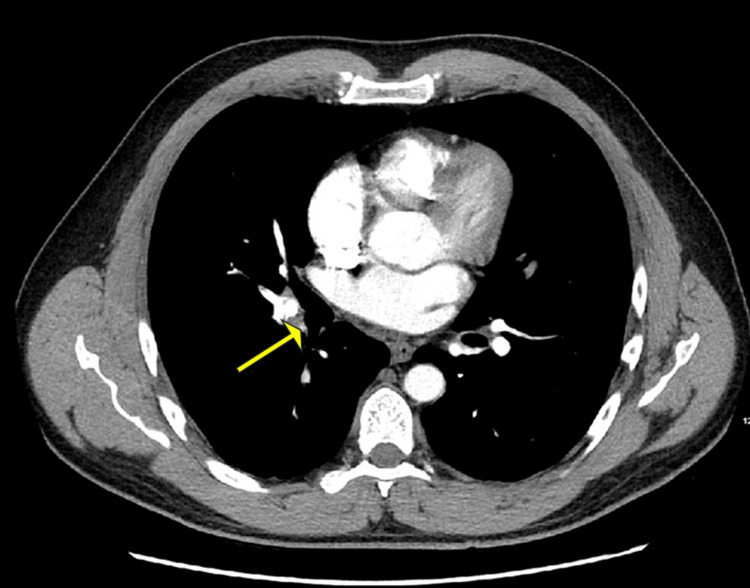
Central filling defect in the ascending interlobar right pulmonary artery branch

**Figure 3:**
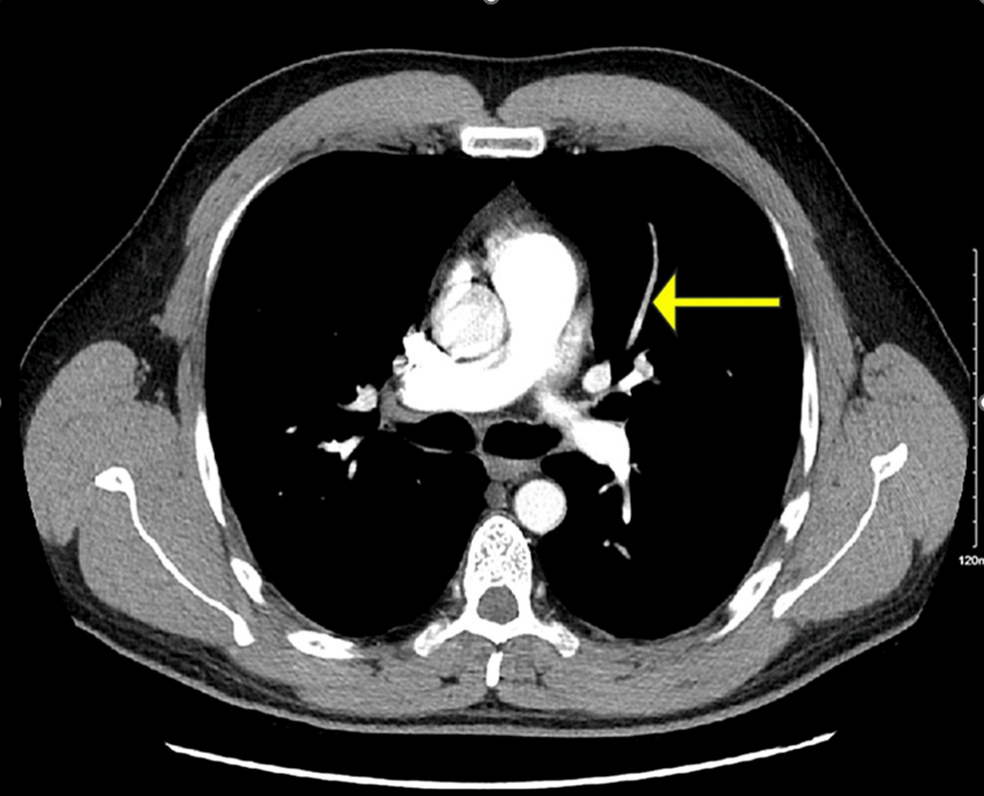
Filling defect in left upper lobe segmental pulmonary artery branches

The lower extremity venous duplex revealed a new noncompressible thrombus located in the left popliteal vein (Figure 1) with no significant color Doppler seen within the vein. Another noncompressible thrombus was seen within the left posterior tibial vein. CT angiography with contrast of the chest revealed bilateral acute pulmonary embolism (PE) (Figures 2-3).

Consequently, the patient was diagnosed with deep vein thrombosis (DVT), PE, and positive LA panel. Therapeutic weight-based low molecular weight heparin (LMWH) was started inpatient. Upon discharge home, his platelet count improved to 110 x 10^3^/µL and LMWH was prescribed with a subsequent follow-up with outpatient hematology and rheumatology.

## Discussion

Since ES is associated with a variety of conditions such as hematological malignancies, SLE, infections, or primary immune deficiencies, its management and prognosis can be significantly influenced and modified [6]. Thus, it is essential to consider both ES and systemic lupus erythematosus (SLE) to quickly and correctly diagnose patients, as treatment and management for both diseases may vary. Given that ES is determined through a process of exclusion, it's critical to rule out other potential causes of AIHA and ITP purpura, such as HIV, medications, and viral infections [7]. Additionally, to prevent bias in diagnosis, the list of potential differentials should remain comprehensive, including conditions like autoimmune lymphoproliferative syndrome, paroxysmal nocturnal hemoglobinuria, thrombotic thrombocytopenic purpura, hemolytic‐uremic syndrome, antiphospholipid syndrome, Sjögren's syndrome, IgA deficiency, lymphomas, chronic lymphocytic leukemia, and drug-induced AIHA [7].

Generally, ES tends to follow a chronic pattern marked by frequent cycles of relapse and remission, which influences the objectives of treatment [6]. Treatment strategies for ES have largely been adapted from those used for isolated autoimmune cytopenia (AIC) [6]. The first-line therapy is corticosteroids and IVIGs while the second-line treatment is rituximab, followed by other options such as splenectomy, danazol, and immunosuppressants [1]. When assessing the connection between ES and SLE, it's essential to recognize the distinctions in clinical management. While SLE cases are typically treated with prednisone and rituximab, managing SLE concurrent with ES may require the addition of romiplostim. Therefore, prompt and accurate identification of ES can facilitate the initiation of suitable therapy with immunosuppressive medications, enabling patients to recover faster and restore hematologic function [7].

It is imperative to take into account the potential variations in how this condition may manifest across diverse populations. Lube et al. [8] and Moskop et al. [9] have both delved into the discussion of pediatric SLE accompanied by ES. Lube et al. identified that because ES was a rare and severe manifestation of childhood SLE with the absence of typical lupus manifestations, patients often required hospitalization and IV treatment [8]. Given the challenges in diagnosing SLE in children, healthcare providers should be mindful that ES and SLE can coexist without the classic SLE symptoms. This awareness is crucial for ensuring timely diagnosis and intervention [9]. Furthermore, as stated earlier, much of SLE with associated ES is extrapolated from what is currently known about AIC treatment guidelines. With an increasing number of accurate diagnoses and reports on various treatment modalities, treatment guidelines can be revised accordingly. Kashari et al. [10] outlined in their report the different treatment methods they used to correct their patient’s thrombocytopenia and anemia to no avail. Ultimately, they resorted to eltrombopag, resulting in a significant improvement in platelet count. Consequently, they concluded that eltrombopag was a safe and efficacious option for managing refractory thrombocytopenia in cases of ES associated with SLE [10].

## Conclusions

In conclusion, this case demonstrates the need to take into consideration one crucial aspect in the management of ES is the recognition of associated autoimmune conditions, particularly LA and SLE. The presence of LA in ES patients can significantly contribute to the hypercoagulable state observed in some cases. The association between ES and SLE underscores the importance of a comprehensive assessment for concomitant autoimmune diseases, as both conditions share underlying autoimmune dysregulation. The prompt identification of LA in ES patients is paramount, as it can guide therapeutic decisions and improve clinical outcomes. Management often involves a multidisciplinary approach, integrating immunosuppressive agents to control autoimmune hemolysis and thrombocytopenia, along with anticoagulation therapy to mitigate thrombotic risk. Individualized treatment strategies aim to achieve hematologic remission while minimizing adverse effects and preventing thrombotic complications.
